# Lymphocyte subsets predict mortality in acute paraquat poisoning

**DOI:** 10.17305/bb.2025.11891

**Published:** 2025-02-17

**Authors:** Qian Dong, Huan Xu, Pengjie Xu, Jiang Liu

**Affiliations:** 1 Department of Nephrology, The Affiliated Lihuili Hospital of Ningbo University, Ningbo, China

**Keywords:** Lymphocyte subsets, paraquat, PQ, PQ poisoning, mortality rate

## Abstract

Paraquat (PQ) is a highly effective herbicide widely used in agricultural production, known for its strong herbicidal power, rapid action, and minimal environmental pollution. However, it is also highly toxic to humans and animals, with acute lung injury (ALI) being the primary cause of death. While the toxic mechanisms of PQ have been studied from various perspectives, its effects on lymphocytes and their subsets remain unclear. This study aimed to explore the relationship between lymphocyte dysfunction and mortality in acute PQ poisoning. A total of 92 patients with PQ poisoning who visited the emergency department of The Affiliated Lihuili Hospital of Ningbo University between January 1, 2016, and September 30, 2021, were included. Basic demographic and laboratory data within 24 h of admission were collected. Peripheral blood lymphocyte subsets were analyzed using flow cytometry. To identify independent risk factors for mortality, patients were followed up for 90 days. COX proportional hazards models and LASSO regression were applied to screen for predictive variables and develop a predictive model. All participants provided informed consent, and the study was approved by the relevant ethics committee. Among the 92 patients, 36 died. Compared with the survival group, the death group showed significantly higher white blood cell and neutrophil counts, lymphocyte counts, and CD4^+^/CD8^+^ T cell ratios, while the percentage of natural killer (NK) cells was significantly lower (*P* < 0.001). COX regression analysis identified these factors as independent risk factors for mortality: lymphocyte count: hazard ratio (HR) ═ 1.59; 95% confidence interval (CI), 1.02–2.47; *P* ═ 0.04 neutrophil count: HR ═ 1.12; 95% CI, 1.06–1.18; *P* ═ 0.04 CD4^+^/CD8^+^ T cell ratio: HR ═ 2.01; 95% CI, 1.03–3.92; *P* ═ 0.04 NK cell percentage: HR ═ 0.88; 95% CI, 0.82–0.95; *P* ═ 0.002. These findings suggest that lymphocyte count, neutrophil count, CD4^+^/CD8^+^ T cell ratio, and NK cell percentage are all associated with mortality in PQ poisoning cases.

## Introduction

Paraquat (PQ), chemically known as 1,1′-dimethyl-4,4′-bipyridinium cation, is a highly effective, non-selective herbicide. Since the 1960s, it has been widely used in agricultural production worldwide, particularly in developing countries [1]. Due to the lack of specific antidotes and effective treatment measures, the mortality rate of PQ poisoning ranges from 50% to 90% [2]. In recent decades, with significant advancements in immunotoxicology, immunotoxicity has become a key factor in evaluating many pesticide compounds [3]. As an organic heterocyclic herbicide, PQ can disrupt immune cell function. However, research on its effects on the immune system remains inconsistent. Some studies suggest that PQ suppresses both cellular and humoral immune functions [4–6], while others propose that it stimulates immune regulation and promotes the expression of inflammatory factors [7–9]. Given these conflicting findings, further research is needed to clarify the immunotoxic effects of PQ on immune cells.

## Materials and methods

### Study subjects

We retrospectively analyzed 114 patients with PQ poisoning who were hospitalized in the Emergency Department of Li Huili Hospital, Ningbo University, between January 2016 and September 2021. The inclusion criteria were: (1) age between 18 and 75 years; (2) a confirmed history of oral PQ ingestion; and (3) a blood or urine PQ concentration of ≥0.1 mg/L. The exclusion criteria were: (1) concurrent poisoning with other pesticides or drugs; (2) a history of blood disorders, tumors, severe infections, or organ dysfunction; and (3) a time interval of more than 48 h between poisoning and hospital admission. Based on these criteria, 92 patients were ultimately included in the study.

### Treatment options

All subjects were given a standardized treatment regimen: (1) General treatment: all patients should be given no water, fasting, and no swallowing saliva immediately; and rinse the mouth with new rehabilitation solution and compound chlorhexidine gargle to keep the mouth clean. If the patient’s arterial partial pressure of oxygen (PO_2_) is greater than 40 mmHg, oxygen therapy is contraindicated. (2) Reduce toxic absorption: all patients should immediately change clothing contaminated with pesticides. Rinse the stomach repeatedly and thoroughly with water, and thoroughly wash the contaminated skin and mucous membranes. (3) Promote the excretion of poisons: all patients were given furosemide to push diuresis. After admission, the patient should immediately take urine for urine colorimetric examination (the colorimetric agent is composed of 2% sodium thiosulfate and 2 mol/L sodium hydroxide, according to the volume ratio of 1:1, stored in a constant temperature refrigerator at −4 ^∘^C), the examination results should be compared with the standard colorimetry, and those with positive urine colorimetry should undergo temporary deep venous catheterization blood purification treatment. (4) Reduce the functional damage of various organs and prevent related complications: all patients were treated with intravenous vitamin C, reduced glutathione and ambroxol hydrochloride antioxidant therapy. Omeprazole is given to inhibit gastric acid secretion, protect gastric mucosa, and prevent gastrointestinal bleeding; ceftazidime is given to prevent infection. Patients with positive urine colorimetry are given intravenous methylprednisolone. (5) Rehydration nutrition support and maintenance of internal environmental homeostasis: according to the patient’s urine output, intravenous fluids ranging from 4000 mL to 6000 mL per day. Electrolytes should be supplemented appropriately according to the patient’s electrolyte profile.

### Research methods

#### Data collection

For each patient, the following data were recorded: name, gender, age, hospitalization number, oral PQ dose, and the time from poisoning to hospital admission. All patients arrived within 48 h of poisoning, and none had undergone self-treatment. Laboratory tests were completed within 24 h of admission—before any therapeutic intervention—including blood routine tests, C-reactive protein (CRP), erythrocyte sedimentation rate (ESR), albumin, aspartate aminotransferase (AST), alanine aminotransferase (ALT), blood urea nitrogen (BUN), serum creatinine (SCr), immunoglobulins (IgG, IgA, IgM), complement (C3, C4), and arterial blood gas analysis. Blood and urine samples were immediately collected for initial urine colorimetric analysis of PQ concentration, followed by precise measurement via high-performance liquid chromatography (HPLC) at the Shanghai Pesticide Research Institute. Upon admission, all patients were assessed using the Acute Physiology and Chronic Health Evaluation (APACHE II) and Sequential Organ Failure Assessment (SOFA) scores. A higher SOFA score indicates more severe organ dysfunction and a higher risk of mortality in PQ poisoning cases. Flow cytometry was used to analyze peripheral blood lymphocyte subsets, including the CD4^+^/CD8^+^ T-cell ratio, B-cell percentage, and natural killer (NK)-cell percentage. Patients were followed up until death or 90 days post-poisoning.

**Figure 1.**
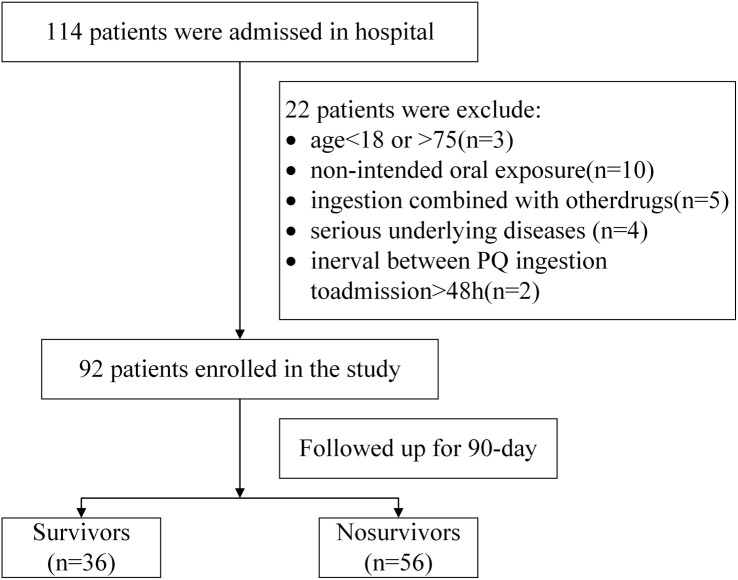
**Flowchart.** PQ: Paraquat.

### Ethical statement

This study was approved by the Institutional Review Board of Li Huili Hospital, Ningbo University (IRB: KY2023SL371-01). Informed written consent was obtained from the patients or their nearest relatives.

### Statistical analysis

SPSS 26.0 statistical software was used for data analysis. Normally distributed data were expressed as mean ± standard deviation (- ± s), and comparisons between two groups were performed using the independent sample *t*-test, while one-way ANOVA was used for comparisons among multiple groups. Non-normally distributed data were expressed as medians and quartiles, with inter-group comparisons conducted using the rank sum test. The chi-square test was applied for analyzing constituent ratios. For survival curve analysis, the Kaplan–Meier method was used. The Cox proportional hazards model was employed to assess whether an indicator was an independent risk factor. Variables with a significance level of *P* < 0.1 in univariate analysis were included in the multivariate risk model for further analysis, and the forward stepwise method was used to eliminate collinearity between variables. A *P* value < 0.05 was considered statistically significant. A nomogram prediction model was developed based on multivariate Cox and LASSO-Cox regression. Calibration curves, decision curves, C-index charts, and ggrisk plots were constructed to evaluate the model’s performance.

## Results

### Patient characteristics

According to the inclusion and exclusion criteria described above, 114 PQ poisoning patients were initially identified, with no cases lost to follow-up. However, 22 patients were excluded for not meeting the criteria, leaving 92 patients for the study (Figure 1). All patients were monitored until death or 90 days post-poisoning. Among them, 56 (60.9%) survived, while 36 (39.1%) died. The cohort included 37 males (mean age: 26.75 years) and 55 females (mean age: 31.50 years). Comparisons between the survival and death groups revealed the following: the death group had significantly higher PQ oral ingestion volume, plasma and urinary PQ concentrations, APACHE II and SOFA scores, WBC and neutrophil counts, CRP, BUN, SCr, AST, and ALT levels than the survival group (*P* < 0.001). Consultation time, ESR, albumin levels, pH value, and partial pressure of carbon dioxide (PCO_2_) differed significantly between the two groups (*P* < 0.05). No significant differences were found in age, gender, hematocrit, red blood cell distribution width, platelet counts, monocyte count, or PO_2_ (*P* > 0.05, Table 1).

**Table 1:** Comparison of baseline and laboratory indicators of patients in the survival group and the death group within 24 h of admission

**Indicators**	**Survival group (*n* ═ 56)**	**Death group (*n* ═ 36)**	***P* value**
Age (years)	35.50 (26.75, 43.00)	31.50 (26.75, 41.25)	0.889
Gender (male/female)	25/31	12/24	0.280
Consultation time (hours)	22.00 (13.00, 30.00)	14.00 (9.38, 20.50)	0.014
Estimated oral PQ ingestion (mL)	10.00 (5.00, 20.00)	68.75 (50.00, 100.00)	<0.001
Plasma PQ concentration (µg /mL)	0.10 (0.10, 0.10)	0.81 (0.44–3.75)	<0.001
Urine PQ concentration (µg /mL)	0.54 (0.10, 2.82)	25.56 (12.04, 69.42)	<0.001
*Score*			
APACHEII	3.00 (2.00, 5.00)	8.00 (5.00, 12.00)	<0.001
SOFA	2.00 (1.00, 4.00)	5.00 (3.75, 8.00)	<0.001
*Complete blood count*			
Hematocrit (%)	38.70 ± 5.26	36.51 ± 5.56	0.060
Red cell distribution width (%)	12.75 (12.38, 13.43)	13.05 (12.60, 13.85)	0.146
Platelets (×10^ˆ9^/L)	146.00 (109.75, 192.75)	138.50 (87.50, 189.25)	0.347
WBC (×10^ˆ9^/L)	10.04 (7.31, 14.00)	22.13 (13.20, 26.30)	<0.001
Neutrophils (×10^ˆ^9/L)	7.84 (6.12, 12.36)	18.37 (11.08, 22.46)	<0.001
Monocytes (×10^ˆ^9/L)	0.40 (0.15, 0.60)	0.36 (0.12, 0.82)	0.952
Lymphocytes (×10^ˆ^9/L)	1.19 (0.66, 1.63)	3.42 (1.55, 4.16)	<0.001
CRP (mg/L)	3.44 (3.30, 6.00)	18.74 (9.22, 52.82)	<0.001
ESR (mm/h)	13.00 (7.00, 19.70)	20.85 (11.00, 26.85)	0.002
*Renal function*			
BUN (mmol/L)	4.30 (3.15, 6.42)	6.90 (5.25, 10.22)	<0.001
SCr (µmol/L)	70.05 (47.85, 102.67)	168.70 (124.05, 208.00)	<0.001
*Liver function*			
Albumin (g/L)	43.55 ± 5.55	40.76 ± 5.33	0.019
AST (U/L)	19.70 (14.90, 25.85)	64.25 (28.85, 339.92)	<0.001
ALT (U/L)	14.40 (9.57, 23.73)	40.40 (14.88, 288.28)	<0.001
*Arterial blood gas analysis*			
PH	7.42 (7.40, 7.44)	7.40 (7.36, 7.43)	0.034
PCO_2_ (mmHg)	36.68 ± 5.61	32.29 ± 8.57	0.004
PO_2_ (mmHg)	91.10 (81.30, 102.25)	83.70 (62.88, 103.50)	0.261

PQ: Paraquat; APACHEII: Acute Physiology and Chronic Health Evaluation; SOFA: Sequential Organ Failure Assessment; CRP: C-reactive protein; ESR: Erythrocyte sedimentation rate; BUN: Blood urea nitrogen; SCr: Serum creatinine; ALT: Alanine aminotransferase; AST: Aspartate aminotransferase; PCO_2_: Partial pressure of carbon dioxide; PO_2:_ Partial pressure of oxygen.

### Relationship between lymphocyte subsets and prognosis

Compared to the survival group, the death group had a significantly higher lymphocyte count (*P* < 0.001), CD4^+^/CD8^+^ T cell ratio (*P* < 0.007), and lymphocyte-to-monocyte ratio (LMR, *P* < 0.004). Conversely, the NK cell percentage and platelet-to-lymphocyte ratio (PLR) were significantly lower (*P* < 0.001). There was no correlation between B cell percentage and mortality (*P* > 0.05, Table 2). Some patients did not complete all immunoglobulin and complement tests within 24 h of admission, leading to incomplete data. To ensure high-quality analysis and minimize selection bias from missing data, we excluded these patients. This allowed us to increase confidence in the study results. Consequently, we performed statistical analysis on 58 patients with complete immunoglobulin data. The results showed no significant differences in IgG, IgA, IgM, C3, or C4 levels between the survival and death groups (*P* > 0.05, Table 3). We incorporated several variables—including neutrophil count, monocyte count, lymphocyte count, CD4^+^/CD8^+^ T cell ratio, B cell percentage, NK cell percentage, PLR, and LMR—into a univariate Cox proportional hazards model. The analysis revealed that neutrophil count, lymphocyte count, CD4^+^/CD8^+^ T cell ratio, B cell percentage, NK cell percentage, PLR, and LMR were all associated with mortality. In the multivariate Cox proportional hazards model, neutrophil count (hazard ratio [HR] ═ 1.12; 95% confidence interval [CI], 1.06–1.18), CD4^+^/CD8^+^ T cell ratio (HR ═ 2.01; 95% CI, 1.03–3.92), NK cell percentage (HR ═ 0.88; 95% CI, 0.82–0.95), and lymphocyte count (HR ═ 1.59; 95% CI, 1.02–2.47) were identified as independent risk factors for death (Table 4).

**Table 2:** Comparison of peripheral blood lymphocytes and their subsets between the survival group and the death group

**Indicators**	**Survival group (*n* ═ 56)**	**Death group (*n* ═ 36)**	***P* value**
Lymphocytes (×10^ˆ^9 /L)	1.2 (0.7, 1.6)	3.4 (1.6, 4.2)	<0.001
CD4^+^	30.2 ± 8.1	33.0 ± 6.9	0.087
CD8^+^	29.0 ± 9.8	25.4 ± 9.7	0.081
CD4^+^/CD8^+^ (%)	1.1 (0.8, 1.4)	1.4 (1.0, 1.8)	0.007
B cells (%)	19.79 ± 7.77	23.31 ± 9.33	0.053
NK cells (%)	15.5 (9.0, 21.0)	7.0 (3.0, 10.2)	<0.001
PLR	128.21 (92.88, 201.07)	49.77 (23.52, 74.18)	<0.001
NLR	7.41 (4.34, 13.88)	6.11 (4.02, 9.11)	0.472
LMR	3.48 (1.77, 6.40)	7.19 (3.16, 25.46)	0.004

NK: Natural killer; PLR: Platelet-to-lymphocyte ratio; LMR: Lymphocyte-to-monocyte ratio.

**Table 3:** Comparison of immunoglobulin and complement characteristics between the survival group and the death group of 58 patients

**Indicators**	**Survival group (*n* ═ 35)**	**Death group (*n* ═ 23)**	***P* value**
IgG (g/L)	11.93 ± 2.50	11.18 ± 3.61	0.353
IgA (g/L)	2.32 ± 0.83	2.46 ± 1.84	0.693
IgM (g/L)	1.36 ± 0.57	1.35 ± 0.95	0.942
C3 (g/L)	1.00 ± 0.23	0.92 ± 0.23	0.205
C4 (g/L)	0.25 ± 0.10	0.22 ± 0.07	0.285

**Table 4:** Relationship between different indicators in the COX model and the risk of death in patients with PQ poisoning

**Indicators**	**Univariate COX model**	**Multivariate COX model**		
	**HR (95%CI)**	***P* value**	**HR (95%CI)**	***P* value**
Neutrophils	1.1 8 (1.1 3–1.24)	<0.001	1.1 2 (1.0 6–1.1 8)	<0.001
Monocytes	1.61 (0.69–3.76)	0.2 74	1.78 (0.58–5.45)	0.312
Lymphocytes	2.57 (1.96–3.36)	<0.001	1.59 (1.02–2.47)	0.04
CD4^+^/CD8^+^	1.72 (1.09–2.70)	0.02	2.01 (1.03–3.92)	0.04
B cells	1.03 (1–1.07)	0.08 2	1 (0.95–1.05)	0.942
NK cells	0.87 (0.8 2–0.92)	<0.001	0.88 (0.82–0.95)	0.002
PLR	0.99 (0.98–0.99)	<0.001	1 (0.99–1.01)	0.706
LMR	1.02(1.01–1.03)	<0.001	1(0.99–1.02)	0.555

NK: Natural killer; PLR: Platelet-to-lymphocyte ratio; LMR: Lymphocyte-to-monocyte ratio.

### Relationship between different indicators in LASSO COX regression and mortality risk in patients with PQ poisoning

**Figure 2.**
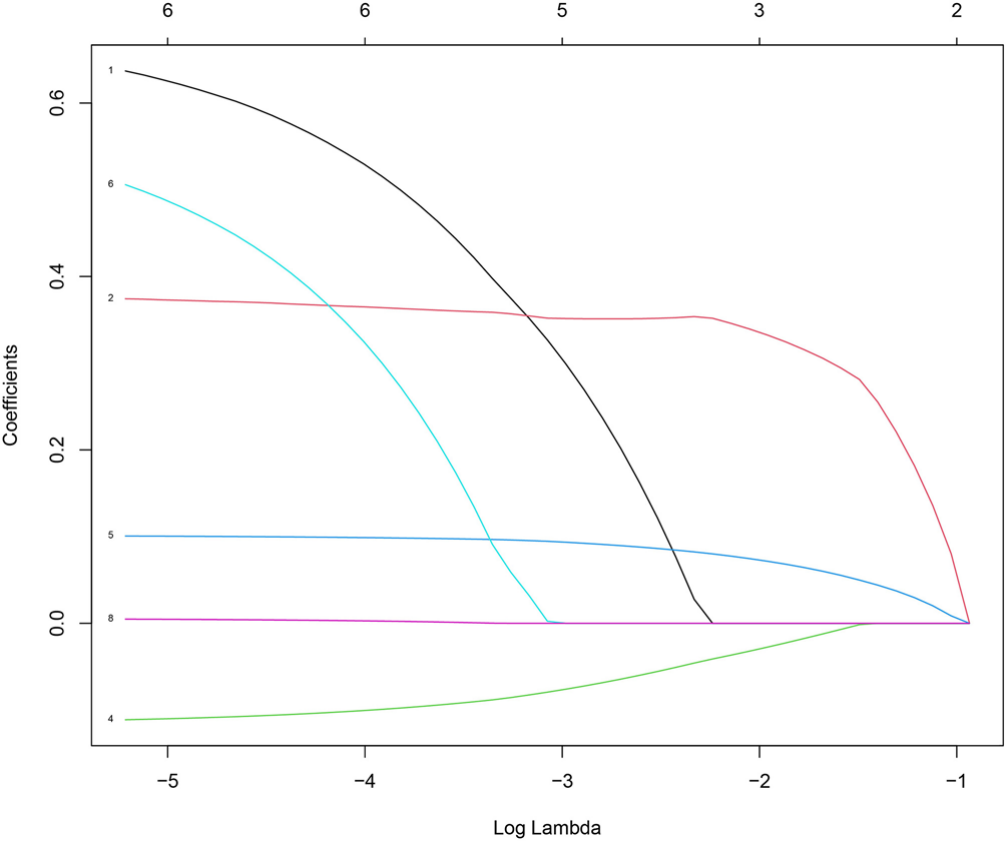
**LASSO coefficient profiles of the eight risk factors.** The lines of different colors represent the situation where the coefficients of different features change with the regularization parameter λ, and as λ increases, more coefficients are compressed to zero, so as to achieve feature selection.

**Figure 3.**
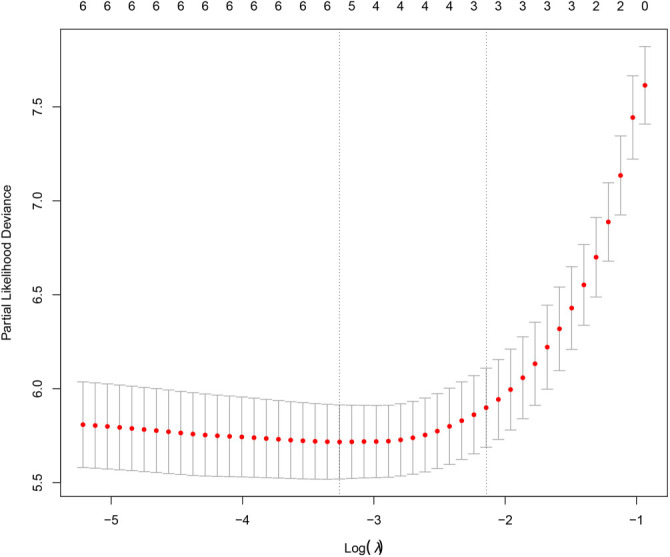
**Three risk factors were selected using LASSO Cox regression analysis.** The two dotted vertical lines were drawn at the optimal scores by minimum criteria and 1-se criteria (at minimum criteria) including CD4/CD8, lymphocytes, NK cells, neutrophils, and monocytes nuclear cells; at 1-s.e. criteria, including lymphocytes, NK cells, and neutrophils.

**Figure 4.**
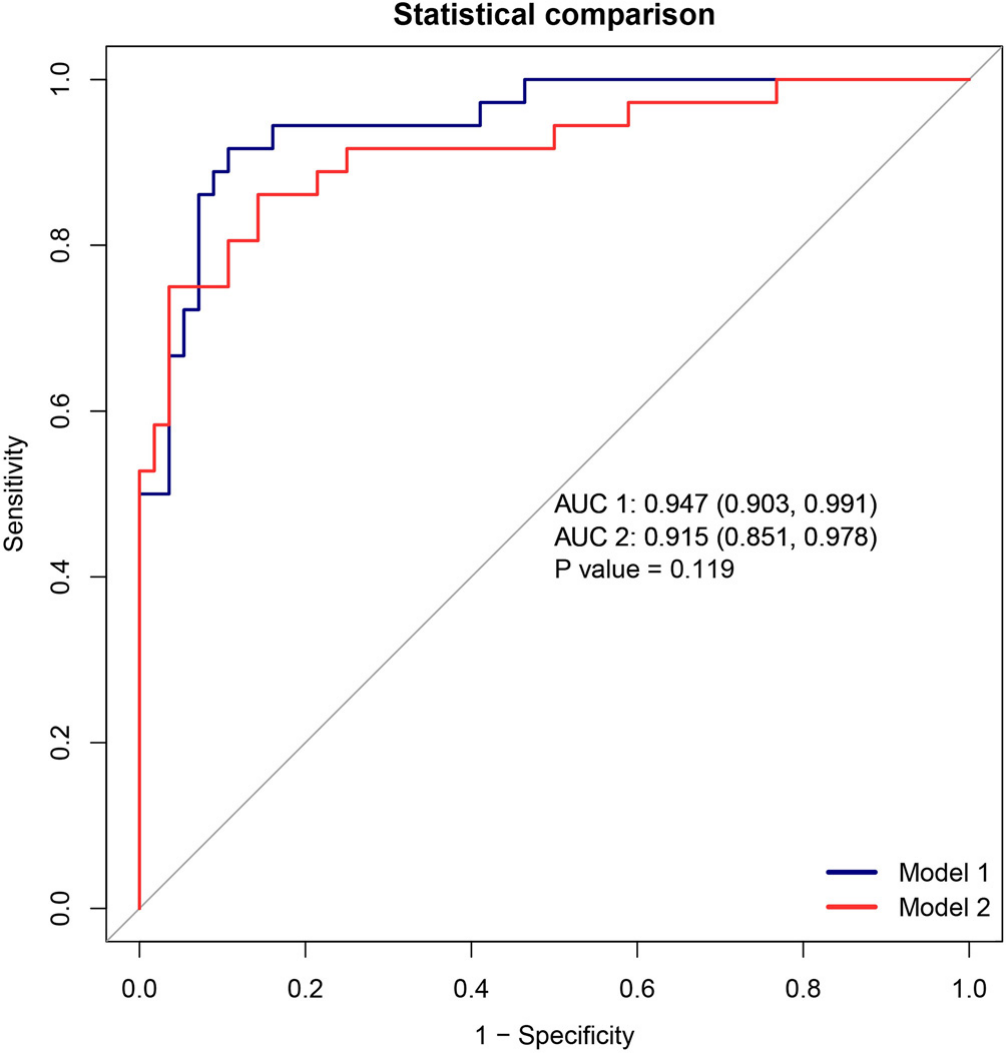
**Model 1 represents the COX regression, and Model 2 represents the LASSO regression.** Both models have an area under the curve value greater than 0.9, indicating good classification performance for predicting mortality in paraquat poisoning patients. However, with a *P* value of 0.119, which is greater than 0.05, it suggests that the diagnostic efficiency of both models is similar.

**Figure 5.**
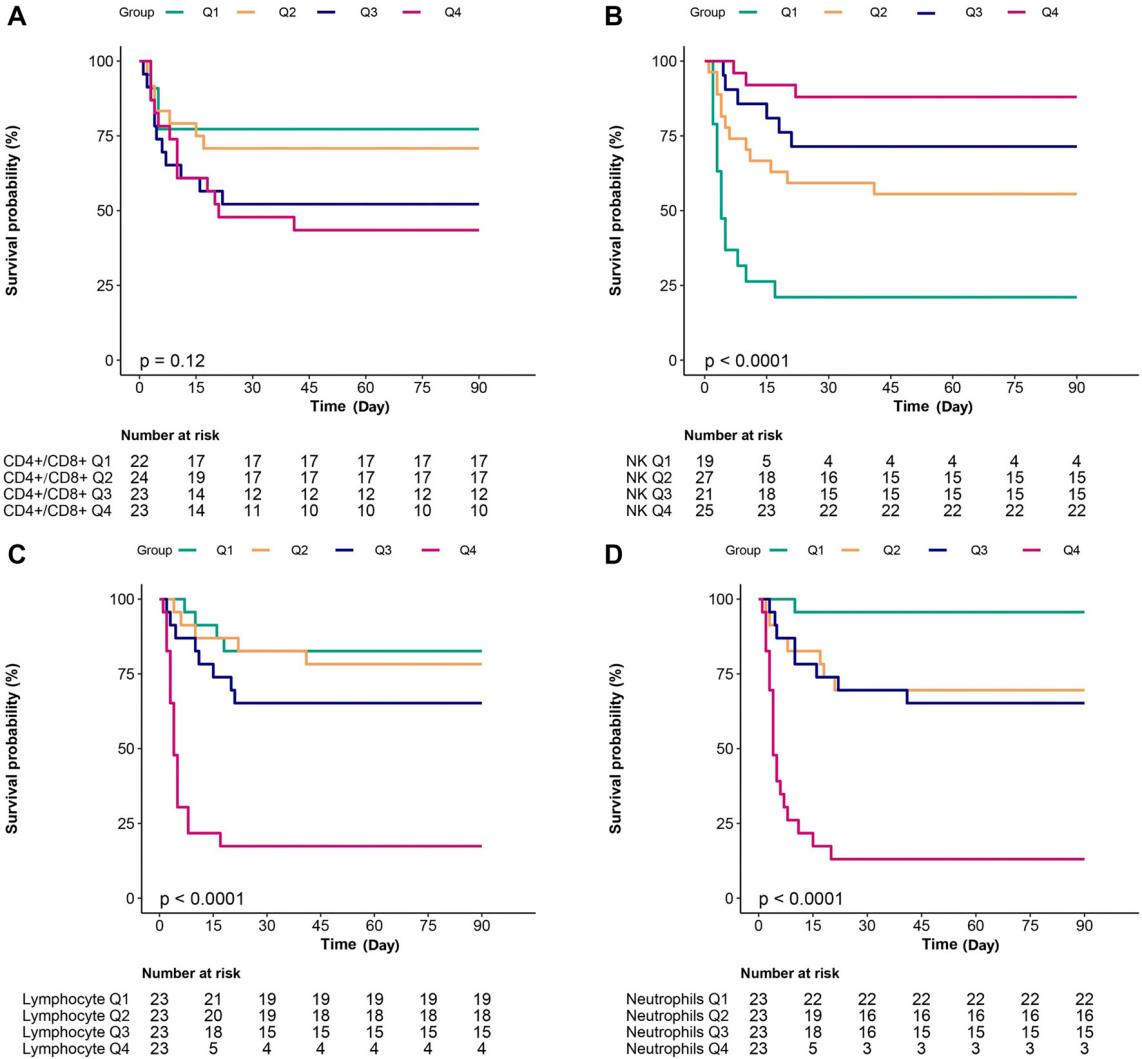
**Comparison of survival time among patients in different quartiles of neutrophil, lymphocytes, CD4^+^/CD8^+^, and NK cells.** Survival curves were plotted using the Kaplan–Meier method for all study subjects, stratified by neutrophil count, lymphocyte count, CD4^+^/CD8^+^ T-cell ratio, and NK cell percentage. The results further confirm that high levels of neutrophil count, lymphocyte count, and low levels of NK cell percentage are indicative of poor prognosis in patients (*P* < 0.001).

**Figure 6.**
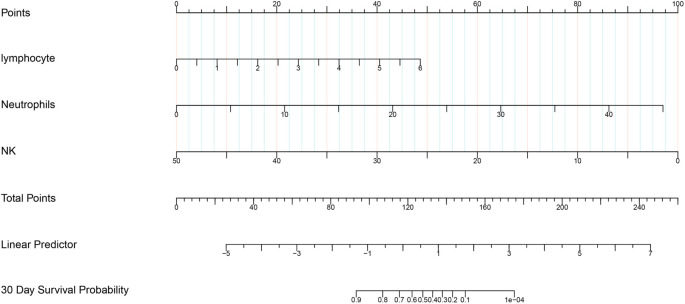
**Nomogram for predicting the survival probability of patients based on lymphocyte count, neutrophil count, and NK cell percentage.** The corresponding points for each variable are determined based on its specific value. The total points are calculated by summing the points for all variables. The 30-day survival probability is then calculated using the Linear Predictor equation: Linear Predictor ═ (0.442 × value lymphocyte) + (0.118 × value Neutrophils) + (−0.109 × value NK).

**Figure 7.**
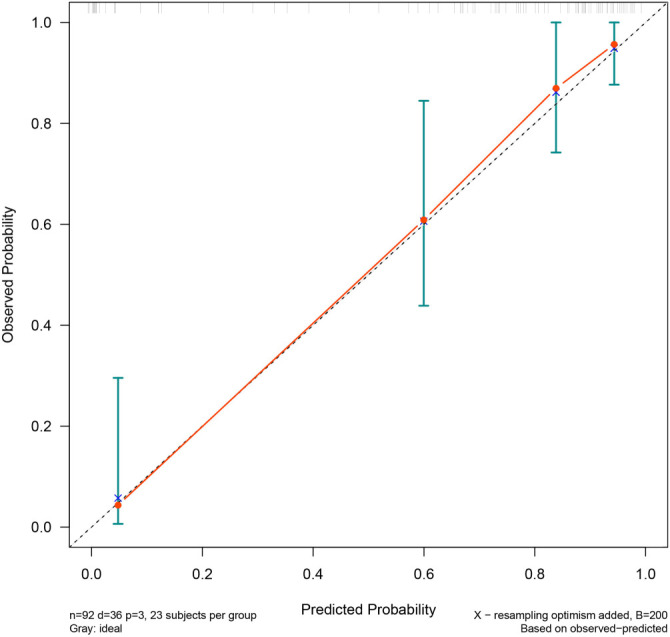
**Calibration curve used to evaluate the calibration ability of the model, which reflects the agreement between the predicted survival probability and the actual observed survival probability.** The gray line represents the ideal scenario, where the model’s predictions align perfectly with the actual results. The blue line represents the calibration curve based on the actual data. This graph illustrates the degree of error between the model’s predictions and actual outcomes, indicating that the model has good calibration.

**Figure 8.**
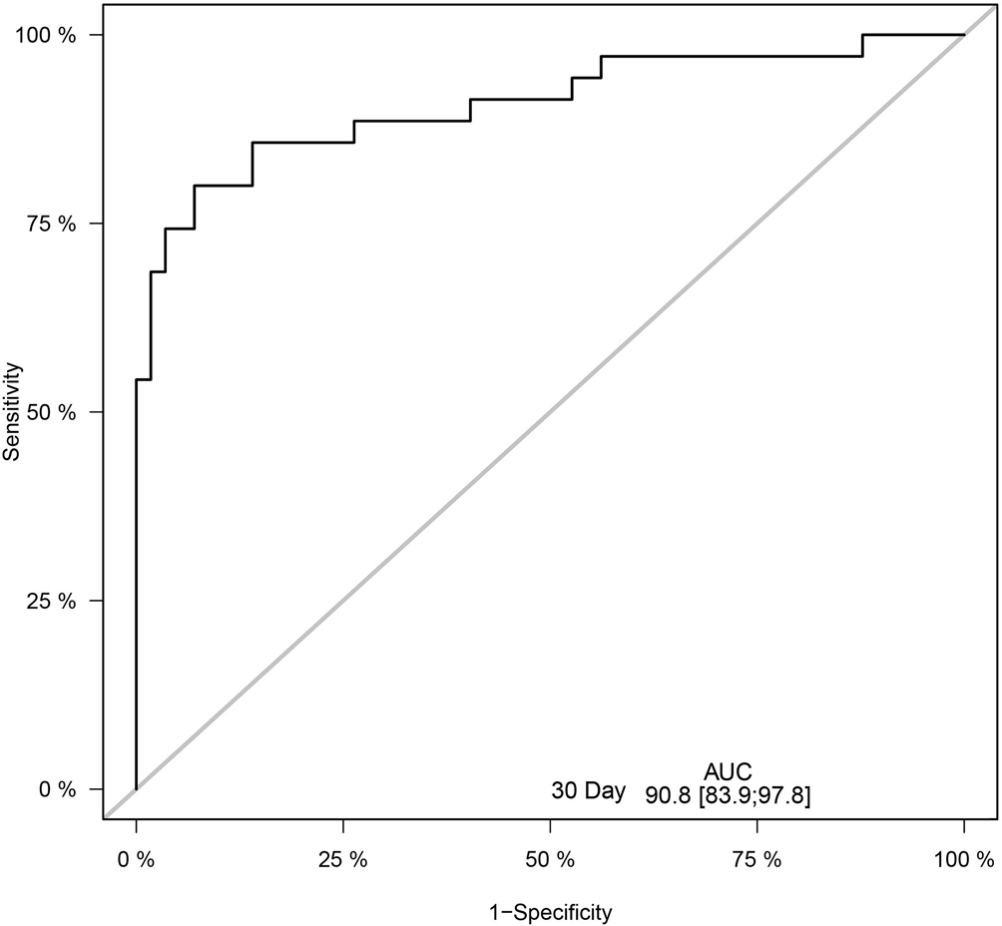
**ROC curve for predicting 30-day mortality.** This curve assesses the discriminative ability of the model, showing the sensitivity and specificity of the model. The AUC is 90.8%, indicating high accuracy in predicting 30-day survival probability (with the AUC ranging from 83.9% to 97.8%). The ROC curve demonstrates the model’s strong discriminatory ability, and an AUC value close to 1 indicates excellent model performance in predicting 30-day survival. AUC: Area under the curve.

We included the eight variables from the previous regression model in the LASSO COX regression analysis (Figure 2). The 1-s.e. standard line indicated that lymphocytes, NK cells, and neutrophils were independent risk factors for the prognosis of PQ poisoning patients (Figure 3). To evaluate predictive performance, we constructed diagnostic ROC curves based on the variables included in both the multivariate COX and LASSO models. The AUC values for both models exceeded 0.9, demonstrating strong predictive capability (*P* ═ 0.119), and suggesting similar diagnostic efficiency between the two (Figure 4). Furthermore, we conducted a survival analysis by categorizing lymphocytes, NK cells, and neutrophils into quartiles. The results showed a significant correlation between these variables and mortality risk in PQ poisoning patients. Specifically, higher lymphocyte and neutrophil levels were associated with an increased risk of mortality, whereas higher NK cell levels were linked to a reduced risk (Figure 5). Based on these findings, we developed a nomogram prediction model for 30-day mortality risk in PQ poisoning patients, incorporating lymphocytes, NK cells, and neutrophils (Figure 6). As a clinical decision-support tool, nomograms offer high applicability, providing clinicians with an intuitive way to quantify risk by integrating multiple prognostic factors. In acute PQ poisoning management, they can help doctors rapidly assess survival probability through emergency blood biochemistry, thereby guiding treatment decisions and resource allocation. The Linear Predictor equation for the model is: Linear Predictor ═ (0.442 × lymphocyte value) + (0.118 × neutrophil value) + (−0.109 × NK cell value). Additionally, a model calibration curve analysis demonstrated strong agreement between predicted and observed probabilities, confirming the model’s high accuracy (Figure 7). The ROC curve’s AUC value of 90.8% further indicated excellent discriminatory ability (Figure 8).

## Discussion

In this study, we statistically analyzed data from 92 patients with PQ poisoning and found significant differences in lymphocyte count, CD4^+^/CD8^+^ T cell ratio, and NK cell percentage between the death and survival groups. More importantly, these immune markers can serve as objective and valuable predictors of acute PQ poisoning outcomes. Our results indicate that lymphocyte count, CD4^+^/CD8^+^ T cell ratio, and NK cell percentage are all associated with mortality in PQ poisoningcases. Lymphocytes play a crucial role in the body’s immune response. PQ poisoning can lead to immune dysfunction, yet few studies have explored its effects on the lymphocyte system. Our findings suggest that absolute lymphocyte count is an independent predictor of death in PQ poisoning, with higher initial lymphocyte levels correlating with increased 90-day mortality. However, the precise mechanism linking lymphocyte count to mortality remains unclear. To better understand this relationship, we further analyzed the characteristics of lymphocyte subsets in PQ-poisoned patients. T cells originate from pluripotent stem cells in the bone marrow, migrate to the thymus for differentiation and maturation under thymosin induction, and then relocate to peripheral lymphoid tissues, where they exert cellular immune functions upon encountering specific antigens. Our study shows that the CD4^+^/CD8^+^ T cell ratio in peripheral blood is positively correlated with mortality following PQ ingestion and can serve as an independent predictor of death. Supporting this, Hassuneh et al. [10] demonstrated in BALB/c mice that PQ activates Th17 cells, leading to increased secretion of interleukin (IL)-21 and IL-9, which further promote Th17 differentiation. Another study [11] found that PQ-poisoned rats exhibited significantly elevated levels of Th17-related cytokines (IL-17, IL-6, and tumor necrosis factor [TGF]-β) in serum, along with increased expression of the key transcription factor RORγt mRNA in lung tissue. These findings suggest that Th17-related cytokines play a role in the pathological process of PQ poisoning. Conversely, IL-17 antibody intervention alleviated lung tissue damage and reduced serum levels of IL-17, IL-6, and TGF-β, as well as RORγt mRNA expression, further confirming the proinflammatory effects of Th17-related cytokines. Additionally, an extensive literature review revealed three reported cases of PQ poisoning in patients infected with human immunodeficiency virus (HIV) [12–14]. Despite receiving standardized treatment, all three patients had good prognoses, which was not attributed to lower PQ intake. A notable commonality among them was their exceptionally low CD4^+^ T cell levels, though the precise reason for their survival remains unclear.

B cells are characterized by the presence of membrane surface immunoglobulin, which functions as a specific antigen receptor (B cell antigen receptor [BCR]). By recognizing different antigen epitopes, BCRs activate B cells, leading to their differentiation into plasma cells and subsequent production of specific antibodies, thereby contributing to humoral immunity [15]. In this study, we found no significant correlation between the percentage of B cells and the mortality rate in patients with PQ poisoning. Additionally, levels of immunoglobulins (IgG, IgA, IgM) and complement components (C3, C4) in 58 PQ-poisoned patients showed no significant association with survival outcomes. These findings are somewhat unexpected given the established role of B cells and immunoglobulins in humoral immunity and the potential impact of PQ on immune function. One possible explanation for this lack of significant changes is the timing of immune parameter measurements. Since immunoglobulin and complement levels were assessed within 24 h of admission, early measurements may not have captured the full extent of humoral immune activation. It is possible that the immune response to PQ toxicity develops later, with immunoglobulin production and complement system activation occurring beyond the initial 24-h window. Another consideration is that PQ toxicity may primarily elicit a cellular immune response rather than a humoral one. Observed changes in T cell subsets—such as an increased CD4/CD8 ratio—and a higher percentage of NK cells suggest that the cellular immune system plays a more prominent role in PQ toxicity pathogenesis. This cellular dominance may explain why changes in B cells and immunoglobulin levels were less pronounced. Further research is needed to elucidate the molecular mechanisms by which PQ affects B cell function and antibody production. In vitro studies and animal models could help clarify PQ’s direct impact on B cells and humoral immunity. Previous research has yielded conflicting results. For instance, Riahi et al. [16] reported that PQ had no significant effect on blood levels of C3, IgM, and IgG, suggesting that PQ does not strongly influence complement proteins or antibody production by B lymphocytes. Conversely, other studies [17] have found that reactive oxygen species (ROS) can significantly reduce IgM production in mouse spleen lymphocytes. Similarly, Hassuneh et al. [10] demonstrated that PQ significantly inhibits B cell proliferation induced by mitogens in vitro, leading to a marked decrease in IgM and IgG levels in mice, while IgA levels remained unaffected. Okabe et al. [18] reported a decrease in IgM and an increase in IgA levels at one, two, and three weeks post-PQ poisoning. The inconsistencies between our findings and those of previous studies may be due to differences in study design. Our research is a clinical retrospective analysis, involving human patients who may have multiple confounding factors, including medication use (e.g., corticosteroids), which could suppress immune responses. Additionally, our study only assessed humoral immune markers within 24 h of admission, leaving open the question of whether PQ poisoning affects humoral immunity at later stages. Future research should explore potential long-term changes in humoral immune function following PQ exposure.

NK cells can directly kill certain tumor cells and virus-infected cells without requiring pre-sensitization by antigen stimulation. As a result, they play a crucial role in the body’s anti-tumor response, early antiviral defense, and immune response to intracellular bacterial infections [19]. Additionally, NK cells activate other immune cells by producing cytokines, such as interferon (IFN)-γ and tumor necrosis factor (TNF)-α, contributing to both innate and adaptive immune regulation [20, 21]. In this study, we found that a low initial percentage of NK cells was associated with 90-day mortality in patients with PQ poisoning and could serve as an independent predictor of death from acute PQ poisoning. This finding aligns with the results of Riahi et al. [4], who reported that PQ inhibits the proliferation of mouse spleen cells in response to the mitogen Phytohemagglutinin-A (PHA), leading to a reduction in NK cell numbers and suppression of cytokine production, including IFN-γ and IL-4. Similarly, Lim et al. [22] demonstrated that NK cell activity in PQ-exposed mice was significantly lower than in the control group, with greater PQ doses causing more pronounced inhibition. Another study [16] found that administering PQ at 1 mg/kg/day significantly impaired mouse spleen cell function and led to a substantial decrease in NK cell numbers. These findings collectively suggest that PQ suppresses NK cell function and reduces their numbers. The mechanism of acute PQ poisoning is not yet fully understood, but five main theories have been proposed: Lipid peroxidation—PQ undergoes single-electron reduction, assisted by NADPH, to form free radicals. These radicals react with molecular oxygen to generate bipyridyl cations and superoxide anions, which are further oxidized to hydrogen peroxide and hydroxyl free radicals. This process induces lipid peroxidation, causing direct damage to essential cellular components [23]. DNA damage—PQ has been shown to induce base modifications and DNA strand breaks. Studies report that PQ exposure leads to sister chromatid exchange, unscheduled DNA synthesis, and positive comet assay results, indicating genotoxic effects [24–26]. Mitochondrial damage—Mitochondria are a primary site of PQ-induced oxygen free radical production. PQ inhibits NADH-Q reductase activity, disrupting mitochondrial electron transferand altering cellular bioenergetics [27]. Additionally, mitochondrial damage causes excessive calcium influx, leading to intracellular calcium overload and exacerbating cellular injury. Enzyme imbalance—PQ poisoning disrupts the balance between tissue inhibitors of metalloproteinases and collagenase, leading to excessive gelatin degradation and apoptosis of alveolar epithelial cells, which contribute to pulmonary fibrosis [28]. Cell activation and cytokine network dysregulation—PQ poisoning triggers infiltration of macrophages, neutrophils, and monocytes in the alveolar cavity and wall. In this study, we also found that both T lymphocytes and NK cells play a role in the pathogenesis of acute PQ poisoning.

Currently, there are few studies, both domestically and internationally, on the effects of PQ on lymphocytes in the human body. Most previous research has been conducted in vitro or through animal experiments. This study is the first to analyze the relationship between lymphocyte subsets and the prognosis of patients with PQ poisoning. However, due to technical limitations, we only measured the relative values of lymphocyte subsets, leaving the relationship between their absolute values and PQ poisoning unclear. Additionally, this study does not fully explain the role of lymphocyte subsets in acute PQ poisoning. Therefore, further in-depth research on the underlying molecular mechanisms is needed.

## Conclusion

In summary, we found the lymphocyte count, neutrophil count, and CD4^+^/CD8^+^ T cell ratio are positively correlated with mortality in paraquat poisoning patients, while the NK cell percentage is negatively correlated with mortality. They may serve as independent predictors of death risk in patients with paraquat poisoning.

## Data Availability

The data that support the findings of this study are available from the corresponding author upon reasonable request.

## References

[1] Dinis-Oliveira RJ, Duarte JA, Sánchez-Navarro A, Remião F, Bastos ML, Carvalho F (2008). Paraquat poisonings: mechanisms of lung toxicity, clinical features, and treatment. Crit Rev Toxicol.

[2] Senarathna L, Eddleston M, Wilks MF, Woollen BH, Tomenson JA, Roberts DM (2009). Prediction of outcome after paraquat poisoning by measurement of the plasma paraquat concentration. QJM.

[3] Jang YJ, Won JH, Back MJ, Fu Z, Jang JM, Ha HC (2015 Sep). Paraquat induces apoptosis through a mitochondria-dependent pathway in RAW264.7 cells. Biomol Ther (Seoul).

[4] Riahi B, Rafatpanah H, Mahmoudi M, Memar B, Brook A, Tabasi N (2010). Immunotoxicity of paraquat after subacute exposure to mice. Food Chem Toxicol.

[5] Shi X, Zhu W, Chen T, Cui W, Li X, Xu S (2022). Paraquat induces apoptosis, programmed necrosis, and immune dysfunction in CIK cells via the PTEN/PI3K/AKT axis. Fish Shellfish Immunol.

[6] Schenker MB, Stoecklin M, Lee K, Lupercio R, Zeballos RJ, Enright P (2004). Pulmonary function and exercise-associated changes with chronic low-level paraquat exposure. Am J Respir Crit Care Med.

[7] Paolillo N, Piccirilli S, Giardina E, Rispoli V, Colica C, Nisticò S (2011). Effects of paraquat and capsaicin on the expression of genes related to inflammatory, immune responses and cell death in immortalized human HaCat keratinocytes. Int J Immunopathol Pharmacol.

[8] Wang Q, Liu S, Hu D, Wang Z, Wang L, Wu T (2016). Identification of apoptosis and macrophage migration events in paraquat-induced oxidative stress using a zebrafish model. Life Sci.

[9] Dinis-Oliveira RJ, de Pinho PG, Santos L, Teixeira H, Magalhães T, Santos A (2009). Postmortem analyses unveil the poor efficacy of decontamination, anti-inflammatory and immunosuppressive therapies in paraquat human intoxications. PLoS One.

[10] Hassuneh MR, Albini MA, Talib WH (2012). Immunotoxicity induced by acute subtoxic doses of paraquat herbicide: implication of shifting cytokine gene expression toward T-helper (T(H))-17 phenotype. Chem Res Toxicol.

[11] Teixeira FFC, Cardoso FGR, Ferreira NS, Corazza BJ, Valera MM, Nascimento GG (2022 Aug). Effects of calcium hydroxide intracanal medications on T helper (Th1, Th2, Th9, Th17, and Tfh) and regulatory T (Treg) cell cytokines in apical periodontitis: a CONSORT RCT. J Endod..

[12] Shang AD, Lu YQ (2015). A case report of severe paraquat poisoning in an HIV-positive patient: an unexpected outcome and inspiration. Medicine (Baltimore).

[13] Tsai JL, Chen CH, Wu MJ, Tsai SF (2016). Paraquat poisoning in patients with HIV infection: a case report and literature review. Medicine (Baltimore).

[14] Ragoucy-Sengler C, Pileire B (1996). Survival after paraquat poisoning in a HIV positive patient. Hum Exp Toxicol.

[15] Catalán D, Mansilla MA, Ferrier A, Soto L, Oleinika K, Aguillón JC (2021). Immunosuppressive mechanisms of regulatory B cells. Front Immunol.

[16] Riahi B, Rafatpanah H, Mahmoudi M, Memar B, Fakhr A, Tabasi N (2011). Evaluation of suppressive effects of paraquat on innate immunity in Balb/c mice. J Immunotoxicol.

[17] Miyazaki Y, Yamasaki M, Mishima H, Mansho K, Tachibana H, Yamada K (2001). Oxidative stress by visible light irradiation suppresses immunoglobulin production in mouse spleen lymphocytes. Biosci Biotechnol Biochem.

[18] Okabe M, Nishimoto S, Sugahara T, Akiyama K, Kakinuma Y (2010). Oral administration of paraquat perturbs immunoglobulin productivity in mouse. J Toxicol Sci.

[19] Quatrini L, Della Chiesa M, Sivori S, Mingari MC, Pende D, Moretta L (2021). Human NK cells, their receptors and function. Eur J Immunol.

[20] Gayoso I, Sanchez-Correa B, Campos C, Alonso C, Pera A, Casado JG (2011). Immunosenescence of human natural killer cells. J Innate Immun.

[21] Di Santo JP (2006). Natural killer cell developmental pathways: a question of balance. Annu Rev Immunol.

[22] Lim JH, Won JH, Ahn KH, Back MJ, Fu Z, Jang JM (2015). Paraquat reduces natural killer cell activity via metallothionein induction. J Immunotoxicol.

[23] Day BJ, Min E, Huang J, Stanley C (2022 Jun 25). The use of thiocyanate formulations to create manganese porphyrin antioxidants that supplement innate immunity. Antioxidants (Basel).

[24] Sofuni T, Ishidate M Jr (1988). Induction of chromosomal aberrations in active oxygen-generating systems. I. effects of paraquat in Chinese hamster cells in culture. Mutat Res.

[25] Ali S, Jain SK, Abdulla M, Athar M (1996). Paraquat induced DNA damage by reactive oxygen species. Biochem Mol Biol Int.

[26] Dusinská M, Kovaciková Z, Vallová B, Collins A (1998). Responses of alveolar macrophages and epithelial type II cells to oxidative DNA damage caused by paraquat. Carcinogenesis.

[27] Tawara T, Fukushima T, Hojo N, Isobe A, Shiwaku K, Setogawa T (1996). Effects of paraquat on mitochondrial electron transport system and catecholamine contents in rat brain. Arch Toxicol.

[28] Ruiz V, Ordóñez RM, Berumen J, Ramírez R, Uhal B, Becerril C (2003). Unbalanced collagenases/TIMP-1 expression and epithelial apoptosis in experimental lung fibrosis. Am J Physiol Lung Cell Mol Physiol.

